# John McCalla

**Published:** 1887-10

**Authors:** 


### Obituary.

John McCalla, D. D. S., died at Millersville, Lancaster county, Pa., July 28, 1887, of heart disease, in the seventy-third year of his age.

Dr. McCalla was born in the province of Ulster, Ireland, November 21, 1814; came with his parents to Philadelphia in 1821. He was apprenticed to the tailoring business, and followed that trade in Philadelphia and Baltimore until 1846, when he commenced the study of dentistry with Dr. C. A. Harris, and graduated at the Baltimore College of Dental Surgery in 1848. He practiced for a short time in Philadelphia; then removed to Lancaster, where he continued to practice until 1877, when he retired from active life and settled in Millerville.

Dr. McCalla was one of the petitioners for the charter of the Pennsylvania College of Dental Surgery, one of the organizers of the Odontographic Society of Pennsylvania, and one of the founders and the first president of the Harris Dental Association. He ranked among the pioneers of progressive dentistry, and was considered as skillful and expert in his profession.—*Dental Cosmos.*

